# Supplementary material for: Bacteriophage-derived endolysins restore antibiotic susceptibility in β-lactam- and macrolide-resistant Streptococcus pneumoniae infections
Source: Mol Med. 2025 May 5;31:170. doi: 10.1186/s10020-025-01226-1 (PMC12051341; doi:10.1186/s10020-025-01226-1)

**Supplementary Information**

**Bacteriophage-derived endolysins restore antibiotic susceptibility in β-lactam- and macrolide-resistant *Streptococcus pneumoniae* infections**

Niels Vander Elst^1^, Kristine Farmen^1^, Lisa Knörr^1^, Lotte Merlijn^1^ and Federico Iovino^1, *^

^1^Department of Neuroscience, Karolinska Institute, Stockholm, Sweden.

*Correspondence to: [federico.iovino@ki.se](mailto:federico.iovino@ki.se)

**Supplementary Figure S1. Comparative analysis of the lytic activity of the cpl-1 and cpl-7s endolysins against the pneumococcal clinical isolates of this study.** cpl-1 and cpl-7s endolysins at 2.5 µM concentration displayed a strong antimicrobial activity towards all pneumococcal isolate of this study in turbidity reduction assays. *S. pneumoniae* AH 16,031 was challenged at a lower OD_620nm_ due to reduced growth *in vitro*. Datapoints show the mean ± standard deviation of three biological replicates (n=3) every 5 minutes.


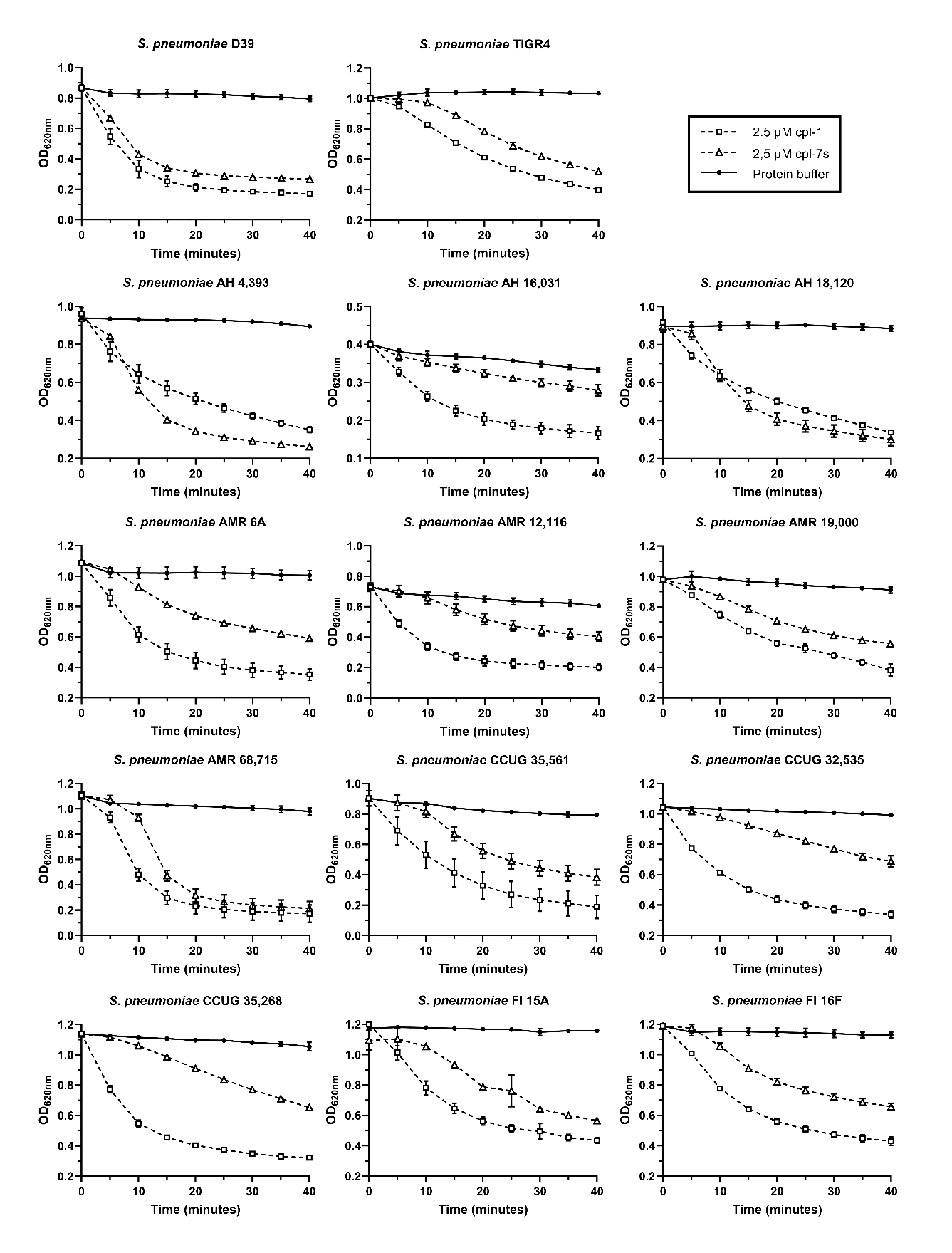


**Supplementary Figure S2. Endolysin cpl-1 crosses the blood-brain barrier (BBB).** (**A**) Western blot analysis of cpl-1 endolysin (detected using an anti-His tag antibody) in samples collected from the lower chamber of a transwell system with a HUVEC monolayer at 30, 60, 90, 120, 240 and 360 min. The medium from the upper chamber (0 min) was used as a positive control. The arrow indicates the band corresponding to cpl-1 endolysin (40.3 kDa). (**B**) Integrity of the HUVEC monolayer was assessed using FITC-Dextran permeability. Fluorescence in the upper chamber (0 min) was compared to the lower chamber at 30, 60, 90, and 120 min to evaluate barrier function. Bars represent the mean of three biological replicates (n=3, shown as white circles), with error bars depicting the standard deviation. (**C**) HUVECs were treated with either phosphate-buffered saline (PBS) or the cpl-1 endolysin, fixed 1 h post-treatment, and stained for lectin (green, AlexaFluor 488) and the intracellular cpl-1 endolysin (red, AlexaFluor 647). Scale bars represent 30.0 µm; *** indicates p < 0.001.

**
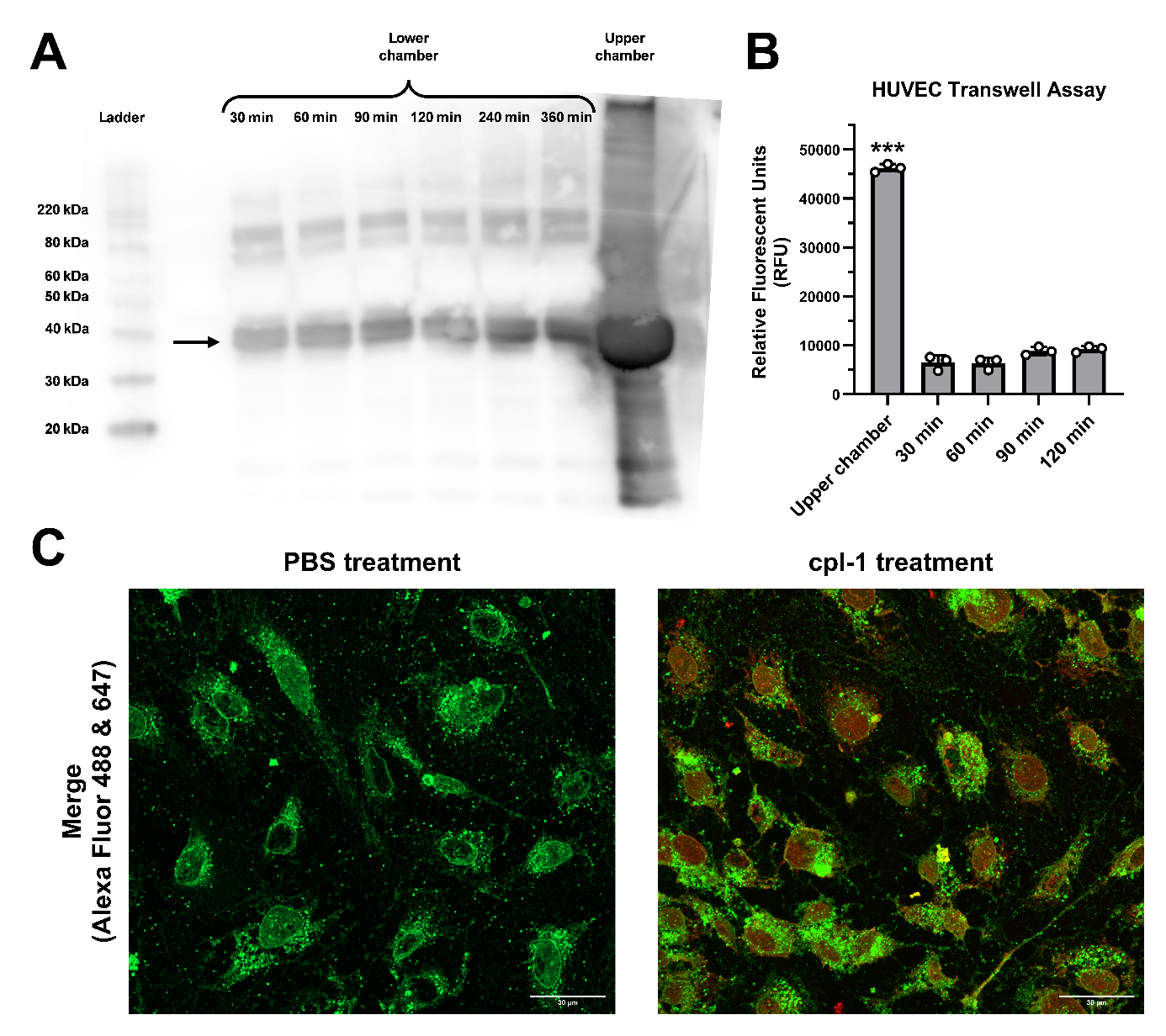
**

**Supplementary Figure S3. Endolysin cpl-1 efficiently crosses the blood-brain barrier.** Confocal images display representative merged z-stacks (20 images, 20.0 µm thickness) of brain tissue sections from mice intravenously administered with PBS (n = 2) or 0.75 mg of the cpl-1 endolysin (n = 2). Tissues were immunofluorescent stained for the brain microvasculature (lectin, Alexa Fluor 488, green signal) and the cpl-1 endolysin (hexahistidine-tag, Alexa Fluor 647, red signal). The lower row depicts merged images that combine the staining for the microvasculature with the cpl-1 endolysin showing no cpl-1 signal for the PBS-treated group, as expected, and diffusion of the red signal from the vasculature into the brain parenchyma for the cpl-1-treated group. Scale bars represent 50 µm.

**
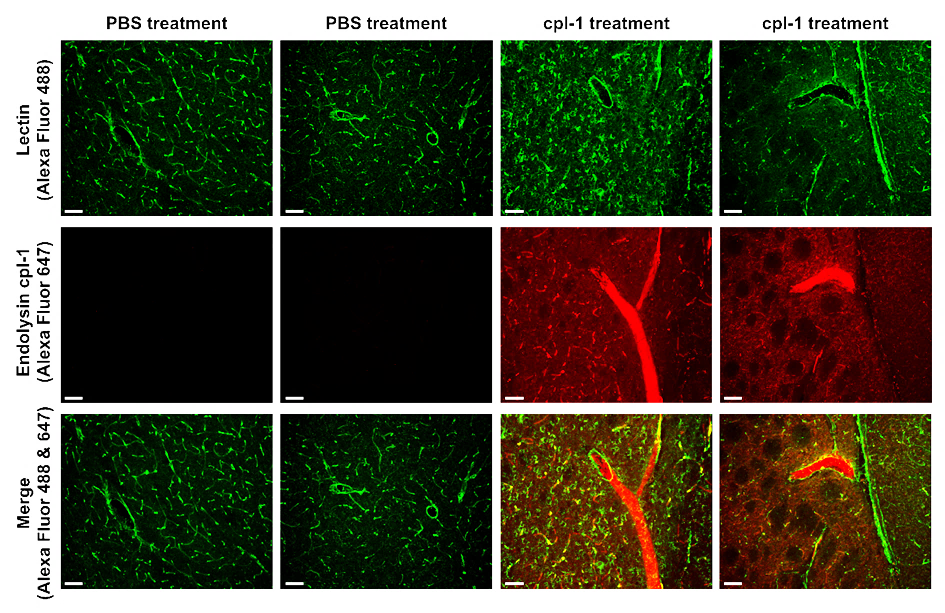
**

**Supplementary Figure S4. *In vivo* validation of a combination therapy with endolysin cpl-1 and penicillin, compared to stand-alone treatments and placebo (PBS) in a penicillin-resistant bacteremia-derived meningitis mouse model.** (**A**) Bacterial load in the spleen. Levels of TNF-α in the (**B**) heart, (**C**) spleen and (**D**) brain, with one mouse from the PBS group excluded due to consistent outlier values. Data were analyzed using one-way ANOVA with Bonferroni post-hoc tests after outlier removal via the ROUT method (Q = 5%); Bars represent the mean ± standard deviation of biological replicates (n=6, 6, 9 and 7 for placebo, endolysin mono-, antibiotic mono- and combination therapy, respectively; with each dot representing one mouse); LOD indicates the limit of detection (200 CFU/mL); *** indicates p < 0.001.


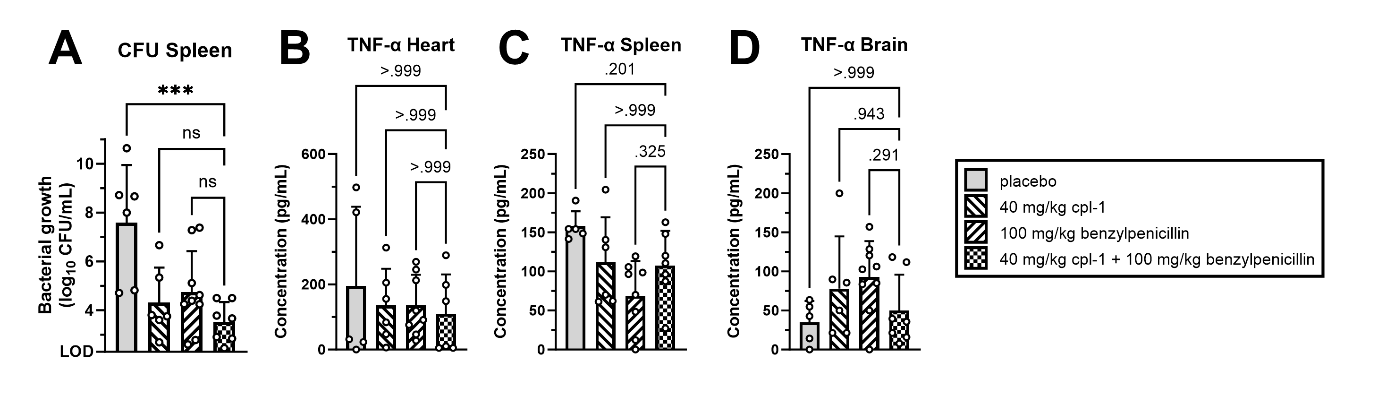


**Supplementary Figure S5. Microglial activation and neuronal damage of a combination therapy with endolysin cpl-1 and penicillin, compared to stand-alone treatments and placebo (PBS) in a penicillin-resistant bacteremia-derived meningitis mouse model.** (**A**) Representative images from each treatment group showing microglial cells (Iba1), and (**B**) cpl-1 treatment increased Iba1 mean intensity compared to placebo treatment. (**C**) Representative images of cortical tissue staining for NeuN (green) and caspase-3 (red) from each treatment group, arrowheads indicate neurons with upregulation of caspase-3 staining. (**D**) Quantification of caspase-3 bright (D) and NeuN positive (E) cells; no significant differences were observed between treatment groups for Data were analyzed using one-way ANOVA with a Bonferroni post-hoc test. Bars represent the mean ± standard deviation of biological replicates (n=3, 3, 6 and 6 for placebo, endolysin mono-, antibiotic mono- and combination therapy, respectively. Scale bars indicate 20.0 µm.


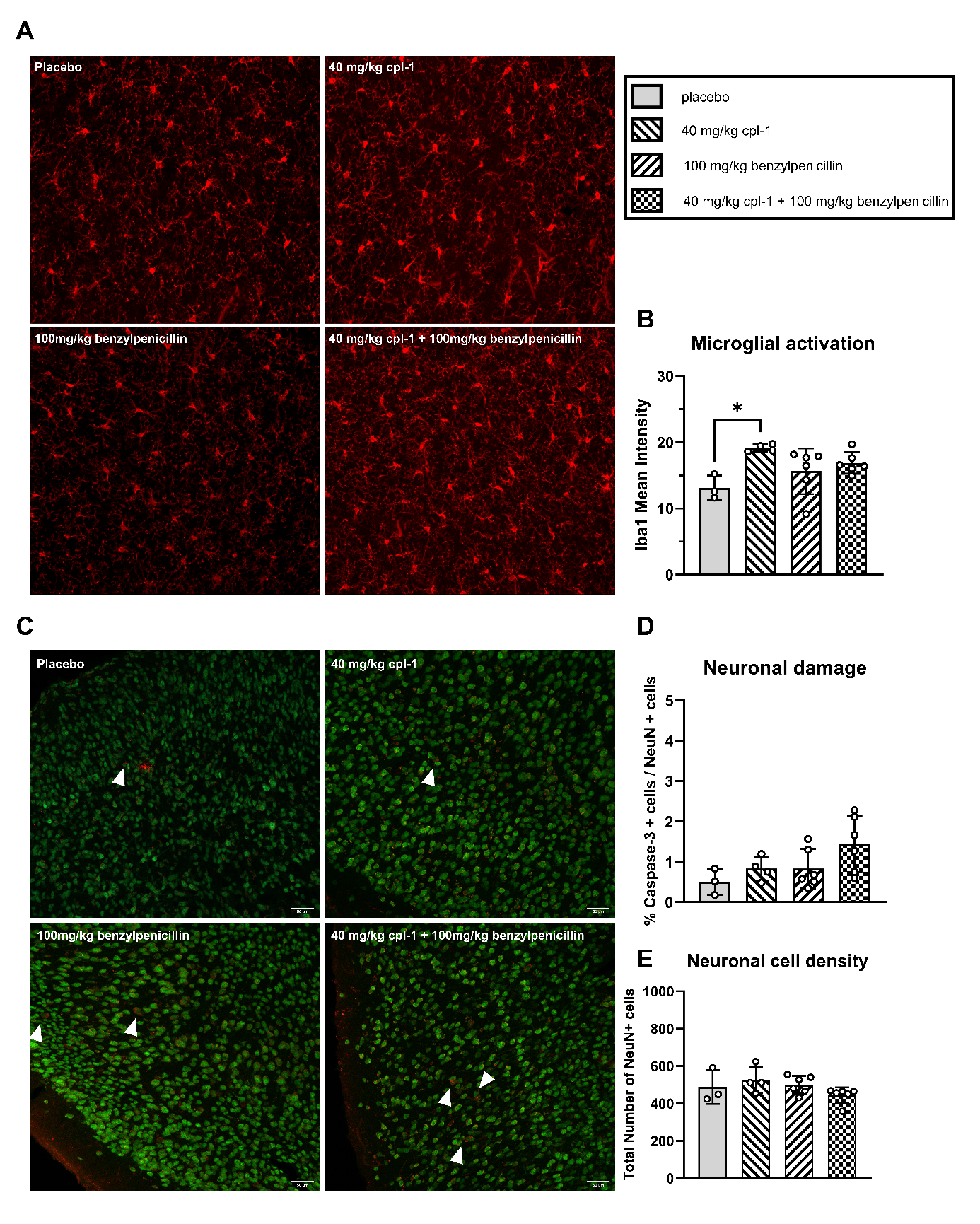

Supplement: Supplementary file 1 — Supplementary Material 1. [file 10020_2025_1226_MOESM1_ESM.docx]
